# Feasibility and acceptability of artemisinin-based combination therapy for the home management of malaria in four African sites

**DOI:** 10.1186/1475-2875-7-6

**Published:** 2008-01-08

**Authors:** Ikeoluwapo O Ajayi, Edmund N Browne, Bertha Garshong, Fred Bateganya, Bidemi Yusuf, Peter Agyei-Baffour, Leticia Doamekpor, Andrew Balyeku, Kaendi Munguti, Simon Cousens, Franco Pagnoni

**Affiliations:** 1 Malaria Research Laboratories, Institute of Medical Research and Training, College of Medicine, University of Ibadan, Nigeria; 2 Department of Community Health, School of Medical Sciences, KNUST, Kumasi, Ghana; 3 Health Research Unit, Ghana Health Service, Accra, Ghana; 4 Department of Sociology, Makerere University, Kampala, Uganda; 5 Institute for Development Studies, College of Humanities and Social Studies, University of Nairobi, Nairobi, Kenya; 6 Infectious Diseases Epidemiology Unit, London School of Hygiene and Tropical Medicine, London, UK; 7 Implementation Research & Methods Unit, UNICEF/UNDP/World Bank/WHO Special Programme for Research and Training in Tropical Diseases (TDR), Geneva, Switzerland

## Abstract

**Background:**

The Home Management of Malaria (HMM) strategy was developed using chloroquine, a now obsolete drug, which has been replaced by artemisinin-based combination therapy (ACT) in health facility settings. Incorporation of ACT in HMM would greatly expand access to effective antimalarial therapy by the populations living in underserved areas in malaria endemic countries. The feasibility and acceptability of incorporating ACT in HMM needs to be evaluated.

**Methods:**

A multi-country study was performed in four district-size sites in Ghana (two sites), Nigeria and Uganda, with populations ranging between 38,000 and 60,000. Community medicine distributors (CMDs) were trained in each village to dispense pre-packaged ACT to febrile children aged 6–59 months, after exclusion of danger signs. A community mobilization campaign accompanied the programme. Artesunate-amodiaquine (AA) was used in Ghana and artemether-lumefantrine (AL) in Nigeria and Uganda. Harmonized qualitative and quantitative data collection methods were used to evaluate CMD performance, caregiver adherence and treatment coverage of febrile children with ACTs obtained from CMDs.

**Results:**

Some 20,000 fever episodes in young children were treated with ACT by CMDs across the four study sites. Cross-sectional surveys identified 2,190 children with fever in the two preceding weeks, of whom 1,289 (59%) were reported to have received ACT from a CMD. Coverage varied from 52% in Nigeria to 75% in Ho District, Ghana. Coverage rates did not appear to vary greatly with the age of the child or with the educational level of the caregiver. A very high proportion of children were reported to have received the first dose on the day of onset or the next day in all four sites (range 86–97%, average 90%). The proportion of children correctly treated in terms of dose and duration was also high (range 74–97%, average 85%). Overall, the proportion of febrile children who received prompt treatment and the correct dose for the assigned duration of treatment ranged from 71% to 87% (average 77%). Almost all caregivers perceived ACT to be effective, and no severe adverse events were reported.

**Conclusion:**

ACTs can be successfully integrated into the HMM strategy.

## Background

Home Management of Malaria (HMM) for children with uncomplicated malaria in high transmission areas in Africa is an integral part of malaria case management within the overall Roll Back Malaria (RBM) strategy [1]. If the Abuja target of 60% of uncomplicated malaria episodes receiving effective treatment within 24 hours [2] and the Millennium Development Targets 5, 8 and 17 (reducing childhood mortality, halting the increase in malaria incidence and providing access to affordable essential drugs in developing countries) [3] are to be met, there is an urgent need to increase access to effective malaria treatment at the community level, especially in underserved rural areas.

So far, 18 African countries have adopted HMM as part of their malaria control programme [4]. Due to unacceptable levels of resistance to chloroquine by *Plasmodium falciparum*, 44 countries in Africa have adopted artemisinin-based combination therapy (ACT) as the first line treatment for uncomplicated malaria [5]. However, in most cases, the deployment of ACT is currently limited to health facilities, with large scale implementation of HMM using ACT delayed by concerns about the use of ACT at the community level [6-8].

A major concern with using ACT at the community level is the potential for poor adherence to the treatment schedule by both caregivers and community medicine distributors (CMDs). This could facilitate the development of parasite resistance to these expensive but currently highly efficacious drugs. Other concerns relate to acceptability by the community, the incidence of adverse events, cost and ability to provide adequate storage conditions to ensure drug stability in the community.

A pilot study carried out in Ghana in 2004 [9] provided preliminary evidence for the feasibility and acceptability of the use of artemether-lumefantrine (AL) in the HMM context. However, this study was essentially a qualitative assessment of community acceptability. To provide additional evidence to support the use of ACT at the community level, the UNICEF-UNDP-WORLD BANK-WHO Special Programme for Research and Training in Tropical Diseases (TDR) funded four studies in sites in Ghana, Nigeria and Uganda, representing different health system and epidemiological settings. This paper reports on the feasibility, acceptability and utilization of ACT provided at the community level.

## Methods

### Study sites and population

This multi-country study was performed in Ghana (two sites), Nigeria and Uganda all in sub-Saharan Africa. In Ghana two districts, Ejisu-Juaben and Ho were studied and in Ho district, an urban and a rural site were studied. In Nigeria, two districts Badeku and Ojoku/Ajia in Ona-ara local government were studied. The sites in Ejisu-Juaben, Ghana; Ona-ara Local Government, Nigeria; and Buguri and Iganga districts, Uganda were all rural areas. Ghana and Nigeria are located in West Africa, while Uganda is in East Africa. Table 1 describes the location and population characteristics of the different study sites, details of the community-based medicine distributors (CMDs) deployed, their training and supervision processes, and type, dosing schedule and cost to the end user of the antimalarial drug used.

**Table 1:** Description of study sites and intervention implementation in four sites evaluating home management of malaria with artemisinin combination therapy

	Ejisu – Juaben District, Ghana	Ho District, Ghana	Badeku and Ojoku/Ajia Districts, Nigeria	Bugiri and Iganga Districts, Uganda
Population characteristics	35 rural communities Population c. 38,000 Perennial malaria transmission	49 urban & rural communities Population c. 42,000 Perennial malaria transmission	40 rural communities Population 43,000 Perennial, hyperendemic malaria transmission	56 rural communities Population c. 60,000 Perennial, hyperendemic transmission
Community medicine distributors	54 (51 male) trusted members of community (farmers, teachers, drug sellers), chosen by the community	76 (48 male) trusted members of community (farmers, teachers, drug sellers), chosen by the community	60 (4 male) including drug sellers, health workers and mothers chosen by the communities	118 (62 male) pre-existing cadre of CMDs (chosen by the community)
	5 days of training	2 days of training	2 days of training	2 days of training
	Provided with bicycles, boots & $3.50 monthly	Provided with T-shirts, watches, raincoats, torches & US$8 quarterly	Received commission of 20–30 US cents per pack distributed. Provided with T-shirts, transport reimbursement, Festivity gift, and certificates.	Transport refund of USD 1.16 per meeting. T-shirts, baseball caps, certificates
	Bimonthly supervision by research staff; Monthly supervision by community Health Officers	Monthly supervision by health staff and quarterly meetings	Monthly supervision by research staff	Monthly supervision by health staff
	CMDs were not obliged to follow up caregivers. However, some of them followed up and some gave drugs under direct observation.		No active follow-up of caregivers by CMDs.	Program ran within existing home management system. CMDs actively followed up treated children to establish treatment outcome
Drugs and dosing schedule	Artesunate+Amodiaquine (ASAQ) once daily for 3 days 2 types of blister pack, for children < 1 year and children 1 year and above		Artemether-lumefantrine (Coartem^®^) twice daily for 3 days 2 types of blister pack, for children < 3 years and children 3 years and above	
	10 US cents for <1 year 20 US cents for 1 year and above		20 US cents for < 3 years 30 US cents for 3 years and above Charged for first 6 months only, thereafter free	Free

### Study design

The study was conducted in three phases. The first phase involved advocacy, community mobilization, selection of CMDs and establishment of key baseline indicators. Information, Education and Communication (IEC) materials were developed and research staff recruited and trained. The intervention phase involved the training of CMDs and distribution of ACT. A year after commencement of ACT distribution, a household survey, interviewing eligible caregivers about child fevers in the past two weeks, was performed to evaluate the intervention.

### Sample size

It was decided that to yield useful results in terms of feasibility, the study should cover populations with a minimum population of around 40,000 – the size of a typical district. The sample size for the household survey was calculated using country-specific fever prevalence rates to provide a precision of ± 5% for the estimates of coverage of ACT treatment through CMDs, assuming a design effect of two. The minimum number of households to be interviewed ranged from 700 to 768.

### Data collection methods

Qualitative and quantitative data collection methods were used. Data collection tools for all the sites were harmonized prior to the intervention with some country specific modifications. The tools included the survey questionnaire and focus group discussion (FGD) and key informant interview (KII) guides, which were pre-tested and translated into the local languages. The format of CMDs' registers was also harmonized. The household survey focused on the health-seeking behaviour of caregivers of children with fever in the preceding two weeks, with an emphasis on timeliness of treatment, compliance with the prescribed treatment course, and perceptions of effectiveness and drug related adverse events. The FGD and KII guides focused on health-seeking behaviours, beliefs about the aetiology of malaria, treatment practices and community perceptions of the intervention.

### Community medicine distributors

Community medicine distributors were trusted members of the community who were chosen by the community from a range of backgrounds, including farmers, chemical/medicine sellers, teachers, traders, community health workers, artisans, 'mother trainers', and opinion leaders (Table 1). In Ghana and Nigeria nurses and community health officers at first-level formal health facilities were counted as CMDs. 'Mother trainers' were lay mothers who were selected from within the communities and trained to distribute drugs.

Selection criteria for CMDs included being a permanent resident (at least one year), trusted and respected by the community, able to keep simple records, and a willingness to serve. Uganda had a functioning pre-existing cadre of CMDs who were co-opted into the study. The number of CMDs per community depended on the community's population size. An average of two CMDs per community was used across the four sites (one CMD per 600 population).

Although CMDs were not paid a salary, various motivation mechanisms were used. Some of these included rain coats, bicycles, boots, watches, T-shirts and certificates of participation. Quarterly monetary allowances varied between USD 4.5 (Uganda), USD 5 (Ejisu-Juaben) and USD 8 (Ho). In Nigeria, the CMDs received a commission of between USD 0.20 and USD 0.30 for each pack of AL dispensed in addition to periodic gifts and reimbursement of transportation fares.

### Drugs used in the study

Two different ACTs were used in accordance with the national drug policies of the participating countries; AL was used in Uganda and Nigeria, and artesunate-amodiaquine (AS/AQ) was used in Ghana.

For the AS/AQ combination, blister packs containing co-packed artesunate and amodiaquine tablets of 50 and 153 mgs respectively were used. For children aged 12 to 59 months the recommended dose of AS/AQ was one tablet of each drug once per day. However, for children of six to 11 months of age, tablets had to be broken in half and repackaged to comply with the recommended AS/AQ dose in that age group.

In Nigeria and Uganda, a fixed combination of 20 mg of artemether and 120 mg of lumefantrine (AL) in two types of blister pack, one for children below three years of age and another for children three years and above, were used. The recommended dose for children aged six to 35 months was one tablet twice daily for three days, and for children aged 36 to 59 months two tablets twice daily for three days. Caregivers were advised to administer the drug after meals, preferably fatty food.

### Drug supply

In all the sites, drugs were provided through existing public health structures, at health district or sub-district level. In general, the distribution points for drugs were the local health facilities. In Uganda, CMDs replenished their stock at monthly meetings at health facility level. In Nigeria and Ghana, CMDs replenished their stocks from the health facilities as the need arose. In some instances drugs were delivered by the research team or by health staff during supervisory visits.

### Supervision

Monthly supervision was undertaken in all sites, by health staff or by the research team (Table 1). In each site, supervision included checking drug stocks, their storage conditions, and the CMDs' registers. The research team provided fuel and travel allowances to supervisors. In Nigeria, unscheduled inspections were periodically undertaken.

### Data analysis

EPI info version 6.02, SPSS version 11.0 and STATA version 9.2 were used by the four sites to enter and analyse data. A set of indicators and quantitative analyses to be performed were agreed in discussion with research staff from each site. Each research team then undertook the analysis of their own data using standard statistical methods. The FGD and KII information was transcribed and content analysis performed. From the transcripts and field notes, the responses to questions asked to explore issues of interest were grouped together, coded, categorized and analysed according to emerging themes. The findings were interpreted and reported in the form of narratives.

### Ethics

Ethical approval for conduct of this study was obtained from the WHO Ethics Review Committee and at national level from the appropriate Ethical Review Boards. Informed consent was obtained from community heads, household heads and the caregivers who participated in the study.

## Results

### Utilization of CMDs

Based on the records maintained by the CMDs, some 20,000 fever episodes in young children were treated with ACTs by CMDs across the four study sites (Table 2). The total number of episodes treated varied considerably between sites as did the average number of episodes treated per CMD (from 17 per CMD in Nigeria to 93 in Uganda). The higher number of episodes treated in Uganda may be explained by the fact that home-based management of fevers has been implemented there since 2001. A higher attrition rate of CMDs in Nigeria compared to other study sites may also have contributed to the lower figures recorded there. Slightly more girls (4,928) than boys (4,595) were treated across the three sites in which this information was recorded. In this study, the accurate population denominators were not available. However, taking into account the width of the age groups, utilization appeared to decline somewhat with age (from 3,571 children in the 6-month age band from 6–11 months to 6,627 children in the two year age band from 36–59 months). Utilization was generally prompt with at least half of all children presenting on the day of onset or within 24 hours of onset in all four sites. In the three sites which recorded promptness as "same day" or "next day" 73–90% of children presented on the day of onset or the next day.

**Table 2:** Utilization of CMDs for treatment of children with fever in 4 sites (source: CMD registers)

	Ejisu – Juaben District, Ghana	Ho District, Ghana	Badeku and Ojoku/Ajia Districts, Nigeria	Bugiri and Iganga Districts, Uganda	Totals
Number of CMDs	54	76	60	118	308
Total number febrile episodes in children less than 5 treated with ACTs by CMDs	4522	3958	1044	11039	20563
Number (%) treated by age					
6–11 months	789 (17%)	474 (12%)	99 (9%)	2209 (20%)	3571 (17%)
12–23 months	1531 (34%)	1157 (29%)	186 (18%)	2571 (23%)	5445 (26%)
24–35 months	1082 (24%)	1054 (27%)	236 (23%)	2548 (23%)	4920 (24%)
36–59 months	1120 (25%)	1273 (32%)	523 (50%)	3711 (34%)	6627 (32%)
Number (%) treated by sex					
Female	2458 (54%)	1977 (50%)	493 (47%)	NA	4928 (52%)
Male	2064 (46%)	1980 (50%)	551 (53%)	NA	4595 (48%)
Number (%) treated by promptness of utilization					
Same day	2636 (58%)	2125 (54%)	479 (49%)	5445 (49%) within 24 hours	14124 (69%)
Next day	1886 (42%)	1313 (33%)	240 (24%)		
Later than next day	0 (0%)	520 (13%)	263 (27%)	5594 (51%)	6377 (31%)

### Treatment coverage of febrile episodes by CMDs

Data from the community-based cross-sectional surveys were used to estimate the proportion of febrile children receiving ACT from CMDs. A total of 2,190 children with fever in the two weeks preceding the survey were identified of whom 1,289 (59%) were reported to have received ACT from a CMD (Table 3). Coverage varied from 52% in Nigeria to 75% in Ho District, Ghana (P < 0.001). Coverage rates did not appear to vary greatly with age, except perhaps in Ejisu-Juaben District, Ghana, where coverage appeared lower in the six to 11 months age group than in older age groups (Table 3). Overall, across the four sites, treatment coverage did not appear to vary strongly with the educational level of the caregiver.

**Table 3:** Treatment coverage of febrile children aged 6–59 months with ACTs obtained from CMDs (source: household survey)

	Ejisu – Juaben District, Ghana	Ho District, Ghana	Badeku and Ojoku/Ajia Districts, Nigeria	Bugiri and Iganga Districts, Uganda	Totals
Total number of febrile children identified	428	124	551	1087	2190
Number (%) treated with ACTs from a CMD	289 (68%)	93 (75%)	288 (52%)	619 (57%)	1289 (59%)
95% CI	61–74%	63–87%	44–61%	51–63%	
Number treated (% coverage) by age in months					
6–11	20 (45%)	13 (76%)	17 (53%)	16 (52%)	66(53%)
12–23	89 (74%)	21 (81%)	49 (49%)	179 (56%)	338 (60%)
24–35	64 (73%)	25 (76%)	69 (52%)	142 (56%)	300 (59%)
36–59	116 (66%)	28 (72%)	153 (54%)	282 (58%)	579 (59%)
Number treated (% coverage) by sex					
Female	137 (65%)	46 (72%)	154 (54%)	NA	337 (60%)
Male	152 (70%)	47 (78%)	134 (50%)	NA	333 (61%)
Number treated (%) coverage by educational level of caregiver					
None	71 (75%)	20 (77%)	80 (47%)	192 (54%)	363 (56%)
Primary	74 (68%)	28 (80%)	155 (56%)	315 (56%)	572 (58%)
Secondary	144 (64%)	45 (76%)	53 (52%)	112 (65%)	354 (63%)
Number treated (% coverage) by marital status of caregiver					
Married	219 (67%)	87 (75%)	256 (54%)	571 (56%)	914 (47%)
Not married	70 (69%)	6 (75%)	30 (41%)	48 (63%)	127 (49%)

### CMD performance

Based on the information recorded by the CMDs in their registers, a very high proportion of children received the correct dose of ACTs (97% or greater in all sites; Table 4). From the survey data, CMDs were reported to have explained the dosing schedule on a very high proportion of occasions (>90% in all sites), but performed less well with respect to explaining danger signs or possible adverse events (Table 4). This was particularly pronounced in Ejisu-Juaben District in Ghana. Availability of CMDs was reported to be good, with 85% or more of caretakers reporting that they found the CMD at the first time of visiting.

**Table 4:** Measures of CMD performance in delivering ACTs in 4 sites (source: CMD registers + household survey)

	Ejisu – Juaben District, Ghana	Ho District, Ghana	Badeku and Ojoku/Ajia Districts, Nigeria	Bugiri and Iganga Districts, Uganda	Totals
Correctness of prescription (from CMD register)					
Number (%) of all children correctly dosed	4473 (99%)	3900 (98%)	1019 (98%)	10,671 (97%)	20063 (98%)
Number (%) of young children receiving an over dose	30 (0.7%)	15 (3.2%)	11 (2%)	27 (0.4%)	83 (0.7%)
Number (%) of older children receiving an under dose	8 (0.2%)	43 (1.2%)	12 (3%)	54 (1.5%)	117 (1%)
Number (%) of occasions on which CMD explained (from survey):					
Treatment schedule	281 (97%)	93 (100%)	264 (92%)	569 (92%)	1207 (94%)
Danger signs	53 (18%)	83 (89%)	240 (83%)	462 (75%)	838 (65%)
Possible adverse events	44 (15%)	86 (93%)	241 (84%)	N/A	371 (55%)
Availability of CMDs (from survey)					
Number (%) of mothers who did not find the CMD at the first attempt	36 (12%)	5 (5%)	36 (12.5%)	86 (14%)	163 (12.5%)
Storage of ACTs (from supervision records)					
Number (%) of supervisory visits at which drugs were stored appropriately	523 (99%)	720 (100%)	960 (100%)	420 (100%)	2623 (99.8%)
Attrition of CMDs					
Number (%) of CMDs withdrawing from role	0 (0%)	3 (4%)	6 (10%)	0 (0%)	9 (3%)

A young woman in Nigeria said:

*'CMDs are always available. It is good we have two in our community, when one is out the village the other one attends to caregivers'*.

In two of the four sites there was some turnover of CMDs but the attrition rate in these sites was relatively low. The need for incentives for CMDs, however, was often mentioned as an issue. An opinion leader in Ho, Ghana, said:

*There is this saying that "the one who feeds a child will surely put her hands in her mouth"; our volunteers should be appreciated so that they can enjoy the work they are doing. For this, the community and I will think about it and compensate them by giving some allowance because sometimes they have to go and call them from wherever they are*.

### Caregiver adherence

From the survey data, a very high proportion of children were reported to have been treated promptly in all four sites (Table 5). The proportion of febrile children who received prompt treatment and received the correct dose for the assigned duration of treatment ranged from 71% in Uganda to 87% in Ghana (P < 0.001; Table 5). Adherence by caregivers was linked to better treatment outcome. A woman participating in a FGD in Ejisu-Juaben district said:

**Table 5:** Adherence of caregivers to treatment schedule

	Ejisu – Juaben District, Ghana	Ho District, Ghana	Badeku and Ojoku/Ajia Districts, Nigeria	Bugiri and Iganga Districts, Uganda	Totals
Number of episodes treated with ACTs from a CMD	289	93	288	619	1289
Number (%) of children correctly treated (dose and duration)	281 (97%)	69 (74%)	256 (89%)	490 (79%)	1096 (85%)
Number (%) of children treated promptly (receiving first dose on the same or next day)	259 (90%)	89 (96%)	278 (97%)	531 (86%)	1157 (90%)
Number (%) of children treated promptly AND correctly	252 (87%)	69 (74%)	231 (80%)	438 (71%)	990 (77%)

*"When I went in for the drug he gave me instructions on how to give the drug to the child. I was told to give it to him continuously for three days – two tablets (white & yellow) each day. I did that and afterwards the child was well again. Happiness was then restored in my home"*.

A local council chairperson of Bwalula village in Uganda explained:

*From 1–2 weeks during Homapak, it now takes about three months before my child gets sick. It is better than CQ when you complete the dose*.

### Safety and perceived effectiveness of ACTs

The proportion of children for whom an adverse event was reported to the CMD was low (circa 1%) in both sites in which this information was available (Table 6). At the survey the proportion of children reported to have experienced an adverse event following treatment with ACT was somewhat higher (4–8%) but none of these adverse events were reported to be serious. In all sites almost all caretakers perceived ACT to be effective. In Ho, Ghana, a community member argued that:

**Table 6:** Safety and perceived effectiveness of treatment of ACTs obtained from CMDs

	Ejisu – Juaben District, Ghana	Ho District, Ghana	Badeku and Ojoku/Ajia Districts, Nigeria	Bugiri and Iganga Districts, Uganda	Totals
CMD registers					
Number of episodes treated with ACTs from a CMD (CMD registers)	4522	3958	1044	11039	20563
Number (%) of children reporting an adverse event to a CMD	63 (1%)	10 (<1%)	NA	NA	
Number (%) children reported recovered	NA	NA	NA	NA	
					
Survey data					
Number of episodes treated with ACTs from a CMD (surveys)	289	93	288	619	1289
Number (%) of children reporting an adverse event at survey	24 (8%)	7 (8%)	10 (4%)	34 (6%)	75 (6%)
Number (%) children reported recovered	NA	NA	284 (99%)	NA	284 (99%)
Number of caregivers (%) who perceived treatment to be effective	286 (99%)	93 (100%)	280 (97%)	NA	659 (98%)

*"The change is that, previously, when children fall sick, we take some time to look for money and walk all the distance to go to the hospital. Sometimes before we get there the child's condition becomes worse. If you are lucky to get to the hospital early, after treatment you walk the same distance back or referred to the big hospital. The unlucky ones either die before they get to the clinic or die at the clinic; but ever since the introduction of this program, we have seen a change. For a year now, since the introduction of the new drug, no child has died and we have not sent any of them to the hospital"*.

A caregiver added:

"As for the drug, it works like magic!"

## Discussion

There are two major findings from this study. First, it showed, at a larger scale than previously [9], that making ACT available at the community level through trained CMDs results in a high degree of adherence by sensitized caregivers. Second, that community-based strategy can deliver high coverage of febrile episodes in children with prompt and adequate treatment. Importantly, these findings are consistent in four different sites in sub-Saharan Africa, both in West and East Africa.

There was high utilization of CMDs. In an estimated population of children aged 6 to 59 months across the sites of 27,450, the CMDs treated 20,563 episodes of fever during the year of implementation (0.75 episodes of fever per child on average)). This contrasts with the figure of 0.12–0.34 episodes per year reported from African health facilities in available literature [10]. Furthermore, the majority of caregivers reported that CMDs were available at the first time of asking, and very little attrition of CMDs was reported. This demonstrates the potential of CMDs to increase access to care in underserved rural areas in Africa, where government health facilities are often under-utilized [11,12]. While every effort was made to ensure the implementation of the study as close as possible to real life conditions, its short duration and the research setting are likely to have contributed to the low attrition rate of CMDs observed in this study. Indeed, the need to provide CMDs with incentives was repeatedly mentioned in interviews and FGDs, and financial motivation is known to be a key factor determining long term performance of CMDs [13]. It is clear that this need must be addressed to ensure the long-term sustainability of CMDs performance.

Concerns have been raised by several authors about the potential misuse of ACT when deployed beyond the health system [6,7], which could lead to wastage of resources and the development of resistance to ACT. However, our findings indicate that there was appropriate prescription in terms of dose by CMDs and correct use by caregivers. In all study sites, 97% or more of the CMDs prescribed the correct dose of ACTs, and almost all of them explained the treatment schedule to the caregiver. Drug storage conditions were satisfactory in all sites.

These studies were designed for the CMDs to provide treatment on the basis of a clinical diagnosis. The possibility of moving away from a symptom based treatment to more complex therapeutic protocols based on parasitological confirmation is currently being investigated. To bring parasitological diagnosis to the community level will require reliable diagnostic tools, and evidence of feasibility and acceptability by the community.

Adherence to the full treatment course has been highlighted as a matter of concern [14-16]. AL has to be taken twice a day for three days (one or two tablets depending on the age group), AS/AQ once daily for three days but two different tablets (or half tablets) each time. Both of these regimens are different from those for older antimalarials like chloroquine or sulphadoxine-pyrimethamine (SP). Nonetheless, reported adherence by caregivers to the correct treatment schedule in terms of dose and duration was high for both treatment regimes in all sites, ranging from 79% in Uganda to 97% in Ejisu, Ghana (85% on average across the sites). These findings are reassuring with regard to the potential for development of resistance, as exposure to sub-therapeutic drug levels is known to be a major factor in the selection of resistant parasites [17].

Furthermore, the majority of caregivers obtaining ACT from a CMD reported that they treated their children promptly (ranging from 86% to 97%, 90% on average across the sites, Table 5). Data from CMDs' registers (Table 2) support this finding, albeit with slightly lower proportions of children recorded to be treated on the day of onset or the next day in three sites. In Uganda, CMDs reported treating only half of the episodes within 24 hours, a proportion which is still substantially higher than previously reported by Nsungwa-Sabiiti [16]. The design of this study does not provide robust data on pharmacovigilance. However, both CMDs and caregivers report a very low incidence of adverse drug effects, none of which were reported to be serious.

Access to prompt and effective treatment is a cornerstone of the current malaria control strategy [18]. Several strategies have been developed to address the issue of access to antimalarials in Africa, including home management of malaria [1], improving the services of private medicine sellers [19], a set of integrated interventions including social marketing approaches [20] and improving the quality of health service performance [21]. Our study provides evidence that once the option of treating children with effective antimalarials close to home is made available to caregivers, a majority of them (ranging from 52% in Nigeria to 75% in Ho, Ghana) make use of it, in most cases in an appropriate and timely manner. This option should be regarded as a complementary to, rather than competing with, health facility delivery of care. As highlighted by Unger *et al *[21], interventions aiming at improving use rates of general health services have also a high potential of increasing malaria cure rates.

Overall effectiveness of drugs distributed at the community level is known to be influenced by several factors [16,22,23], not all of them addressed in this study. However, the high level of correct prescription by CMDs, the promptness of treatment seeking and adherence to the treatment schedule by caregivers, and the coverage (59% on average across the sites) achieved in this study show that some key factors determining the effectiveness of antimalarial treatment can be successfully addressed. Thus, this approach has great potential to contribute to reaching the Abuja target that "*at least 60% of those suffering from malaria have prompt access to, and are able to correctly use, affordable and appropriate treatment within 24 hours of the onset of symptoms*" [2].

Previous studies have shown that use of the correct dose of chloroquine in uncomplicated malaria correlates with improvements in clinical condition [24], and that HMM with chloroquine has been effective in reducing both mortality[25] and severe malaria morbidity [26]. Studies in Uganda and Tanzania [27,28] have recently shown that the high parasitological cure rate of AL is not affected by unsupervised administration under routine clinical practice. A sub-study evaluating parasitological efficacy of the ACTs used in this study is being undertaken and will be reported on separately.

This study shows that ACT use can be successfully integrated in the HMM strategy.

While further studies are necessary to develop mechanisms for ensuring long-term performance of CMDs and to evaluate the effectiveness and safety of ACTs used within the context of HMM programmes, the findings of this study provide evidence to support scaling-up implementation of HMM with ACTs.

## Competing interests

The author(s) declare that they have no competing interests.

## Authors' contributions

All the authors except SC conceived the study; IA, ENB, BG and FB were principal investigators for their respective country's study site and together with FP, LD, BY and AB participated in the research designed and supervised data collection from the field. BY, PAB and SC performed most of the quantitative data analysis while KM analysed the qualitative data. FP, the WHO/TDR HMM research program manager monitored the four sites. All authors contributed to the draft. All authors read and approved the final manuscript.
